# An Enquiry Relative to Bordeu's Hypothesis of the Origin of the Buffy Coat on the Blood

**Published:** 1819-09

**Authors:** 


FOR THE LONDON MEDICAL AND PHYSICAL JOURNAL.

### *An Enquiry relative to* BORDEU'S *Hypothesis of the Origin of the Buffy Coat on the Blood.* By MONSPELIENSIS.

THE prevalent notions respecting the origin of the buffy coat formed on blood drawn from a vein in some diseases, being various and unsatisfactory; and, as a correct knowledge of the nature of this phenomenon would probably lead to very important and beneficial inferences applicable to the practice of medicine; I am led to believe that you will not be averse to the devoting of a few of the pages of your Journal to the investigation of this subject. At present, I merely offer for insertion the hypothesis of Bordeu, which appears to me to merit a greater degree of attention than has hitherto been conferred on it; but, as I may have overlooked some general objection to it, I solicit, through the medium of your Journal, information of such objection, or other remarks respecting it, as may be discerned by any physiologists who may be disposed to concur with me in promoting the investigation I here propose. I therefore defer, for a short time, the advancing of the observations I have to make relative to the hypothesis of Bordeu.

"The sizy fluid which abounds in the blood in several acute and chronic diseases, has long appeared to me," says Bordeu,* "to be a species of plethora, of superabundance, or of cachexy, which I term mucous. It is the result of a return into the blood of the nutritious matter detached by disease from the parts where it was about to become intimately united with the cellular tissue. I have regarded this superabundant sizy fluid as the primary matter of depositions, that of pus, and that of what are termed coctions. We find this matter during critical, and even symptomatic, evacuations; sometimes in the urine, sometimes in the sputa, sometimes in the depositions, and in metastases. We know, when it does not show itself in the excretions at the periods marked for its appearance in acute diseases, that these diseases then become chronic. There are also occasions in which this sizy fluid, far from being superabundant and forming a plethora, is not found in the appropriate proportion; and it is this which establishes a sort of dissolution of the blood, of which I shall hereafter treat."

And, in his *Recherches sur les Maladies Chroniques*, theor. xxx. he says,

"The cellular organ, or mucous tissue, is, then, the seat of inflammation, and the cause of the tumefaction that accompanies it; for it is rare that tumours are formed in parts simply membranous, in which there is no cellular tissue. The cellular organ also furnishes a mucous or gelatinous matter, proper for the formation and the increase of callosities. This matter (originally the mucilaginous part of aliments) is the nutritious fluid which has not yet been converted into laminæ, and which, in many diseases abounds in the blood, not yet being able to be received into the cellular tissue; as the bile abounds in it when not separated by the liver. We observe on this subject, that, as inflammation of the liver does not always produce jaundice, so every affection of the skin, or of the cellular tissue, does not engender a plethora of the nutritious fluid; because a sufficient reflux of it into the blood does not take place. The gelatinous or nutritious fluid, by reason of its superabundance and the facility with which it concretes, is also the cause of the buffy coat, or pellicle, which floats on the blood drawn from a vein; which pellicle is more or less thick and firm, according to the length of time that the blood reposes in the cup. These pellicles are improperly attributed to the heat attending fever,

* *Analyse Medicinale du Sang*, § xxxi.

which is never sufficiently powerful to produce concretion. It is not with more propriety attributed to a morbific humour which often does not exist; for example, in an inflammation occasioned by a ligature made on a part, when the system is in a state of health. There is, then, in almost all diseases a plethora of the nutritious fluid; and the concretions which are formed on the surface of the blood in acute and chronic affections, are nothing else than this same fluid, which has not been permitted to be lodged in the cellular tissue. This nutritious fluid is also the cause of the whiteness of the blood drawn from nurses; a whiteness which has imposed on some physicians, who consider it, especially if there be fever, as the product of a corrupt humour. Lastly, as the milk sometimes reflows from the breasts into the blood, so does the nutritious fluid in the same manner: that is the reason why the blood of some pregnant women has been found of the colour of milk."

In another place (theor. lxxxiv. *op. cit.*) he says,

"It is not rare, in acute and chronic diseases, to see evacuated, at the time of crisis, a great quantity of mucous fluid with the urine. When separated from the urine, it resembles the white of an egg, both in its consistence and the property it has of becoming thickened by fire. I have given it to a dog, who ate it with avidity, as if his instinct had made him discern in it the true matter for nutrition. This matter is, then, the nutritious fluid, which has undergone a little change. I have seen it abound in some patients, and appear in all their excretions. Although in these persons the stomach has executed its functions very well, yet the state of constriction (serrement, *want of expansion*,) and dryness in which their cellular tissue has been, has rendered the circulation and the application of the nutritious fluid impossible. From this it happens that they fall so rapidly into a state of extreme emaciation, and that the blood drawn from them in the access of fever hardly ever fails to show us the buffy coat or mucous pellicle, even though the plethora of the nutritious fluid, from a reflux of it into the blood, has not been present."

In order to render the above passages fully intelligible to those not conversant with the writings of Bordeu, I shall observe that, by the *cellular tissue*, Bordeu designates the basis or elementary structure of all the organs of the body. This he has fully developed in his *Rechérches sur le Tissu Muqueux*; a dissertation which served as a basis and model to Bichât for the most valuable part of his works, his *Traité des Membranes*.

*London; July* 26, 1819.

